# In Vitro Cholesterol Uptake by the Microflora of Selected Kefir Starter Cultures

**DOI:** 10.3390/life14111464

**Published:** 2024-11-12

**Authors:** Małgorzata Ziarno, Dorota Zaręba, Iwona Ścibisz, Mariola Kozłowska

**Affiliations:** 1 Department of Food Technology and Assessment, Institute of Food Science, Warsaw University of Life Sciences—SGGW (WULS–SGGW), Nowoursynowska 159c St., 02-776 Warsaw, Poland; iwona_scibisz@sggw.edu.pl; 2 Professor E. Pijanowski Catering School Complex in Warsaw, 04-110 Warsaw, Poland; dorotazareba@gmail.com; 3 Department of Chemistry, Institute of Food Science, Warsaw University of Life Sciences—SGGW (WULS–SGGW), Nowoursynowska 159c St., 02-787 Warsaw, Poland; mariola_kozlowska@sggw.edu.pl

**Keywords:** kefir, cholesterol uptake, *Lactococcus*, yeast, digestive model, cholesterol-binding capacity, survival rate

## Abstract

Kefir, a fermented milk beverage, is recognized for its potential health benefits, including its cholesterol-lowering properties. This study demonstrated that selected kefir starter cultures, including *Lactococcus* strains and yeasts, significantly reduce cholesterol-binding capacity under simulated gastrointestinal conditions, underscoring the challenges of probiotic delivery. We compared the performance of these cultures under laboratory conditions (growth broths) and simulated digestive juice models. *Lactococcus* strains showed significant differences in cholesterol binding between the two environments, highlighting the limitations of relying solely on laboratory testing. Yeast cultures also exhibited greater cholesterol binding in their native broths, but their survival was limited in digestive models. Our findings suggest that effective probiotic formulations should prioritize strains with high cholesterol-binding capacity and robust survival rates throughout the digestive tract. This study provides valuable insights for future research on the mechanisms behind these functionalities and the potential of kefir yeast strains for use in human digestive models. Our results can be used to inform the development of improved probiotic formulations for cholesterol management.

## 1. Introduction

High blood cholesterol levels are linked to the development of cardiovascular diseases. Scientific evidence suggests that a 1% reduction in the serum cholesterol concentration is associated with an estimated 2–3% reduction in the risk of coronary heart disease. The regular consumption of fermented milk containing appropriate cultures with cholesterol-reducing properties could reduce the coronary heart disease risk by 6%–10% [1]. Lactic acid bacteria, including lactobacilli, *Lactococcus* spp., bifidobacteria, and yeasts, have been demonstrated to bind cholesterol in laboratory studies [2,3,4,5,6,7,8,9].

Kefir, a fermented milk drink traditionally made from cow or goat milk, originates from East Europe and Southwest Asia. It is typically produced using kefir grains or starter cultures that contain the necessary microflora to carry out lactic and alcoholic fermentation, processes that are characteristic of kefir production, including lactic acid bacteria, yeasts, and sometimes acetic acid bacteria [10,11,12,13]. Kefir’s complex microbiota, which comprises both eukaryotic and prokaryotic organisms, is associated with various health benefits. However, kefir formulations exhibit global diversity. Traditional kefir, produced by adding kefir grains to milk, contains yeasts (such as lactose-fermenting yeasts such as *Kluyveromyces marxianus* and non-lactose-fermenting yeasts such as *Saccharomyces unisporus*, *Saccharomyces cerevisiae*, and *Saccharomyces exiguus*), lactic acid bacteria (such as lactobacilli, *Leuconostoc*, and *Lactococcus*), and *Acetobacter*, which all grow in strong symbiosis. Contemporary kefir-type fermented milk drinks, obtained using kefir starter cultures, do not always contain yeasts or may contain only selected yeast groups, such as lactose-fermenting ones, e.g., *K. marxianus* or non-lactose-fermenting ones, e.g., *Saccharomyces*. They may contain only lactic acid bacteria [13]. Kefir is known for its probiotic properties and potential health benefits, including its cholesterol-lowering effects [10,14,15]. The mechanisms behind these effects are not fully understood, but kefir starter cultures are thought to play a role in the binding of cholesterol and the prevention of its absorption in the intestines. However, more clinical evidence is needed to strengthen these proposals [16].

There is scientific evidence that the consumption of fermented dairy products, such as yogurt, can lower cholesterol levels in humans [17,18,19]. Studies on both animals and human volunteers suggest that the presence of lactic acid bacteria in fermented dairy beverages can contribute to reducing blood cholesterol concentrations [20,21]. The cholesterol-lowering activity of lactic acid bacteria is one of their most promising probiotic properties. Some in vitro studies have shown that not only probiotic strains but also some “traditional” lactic acid bacteria used in the production of cheese, cream, butter, or buttermilk may possess cholesterol-lowering properties [2,3,5]. Several hypotheses have been proposed to explain the hypocholesterolemic effects of lactic acid bacteria and bifidobacteria. One hypothesis suggests that bacterial cells bind to cholesterol through attachment via the cell wall (adhesion) or the incorporation of cholesterol into the cell membrane (assimilation) [4,22,23]. Another hypothesis involves the hydrolysis (deconjugation) of bile salts, which necessitates the synthesis of new bile from cholesterol stored in the body, thereby lowering cholesterol levels [24,25,26,27,28]. However, in many cases, the results of in vitro studies are inconclusive or lack reproducibility [19,29]. Lactic acid bacteria could be considered as alternative supplements with health benefits, including cholesterol-lowering effects, for humans. However, the beneficial effects of lactic acid bacteria are dependent on their survival during their passage through the human gastrointestinal tract and their ability to favorably influence the gut microbiota.

The aim of this study was to investigate cholesterol uptake by the microflora contained in selected kefir starter cultures, including both *Lactococcus* and yeast, under in vitro conditions (in growth media) and during their passage through simulated gastrointestinal models. By examining cholesterol uptake under both controlled growth and simulated gastrointestinal conditions, we aim to gain a better understanding of the mechanisms underlying the potential cholesterol-lowering effects of kefir.

## 2. Materials and Methods

### 2.1. Starter Cultures and Materials

The biological material used in this study consisted of lactic acid bacteria isolates from *Lactococcus* species and yeasts from 16 commercial multistrain starter cultures dedicated to kefir production (Table 1). Lactic acid bacteria isolates collected from kefir starter cultures were identified, and only those classified as belonging to the *Lactococcus* genus were selected for further research. Isolation and confirmation of the classification of lactic acid bacteria were conducted using M17 agar (Merck, Darmstadt, Germany) and API CHL tests (bioMérieux, Craponne, France). The API 50CHL test, a kit containing 50 biochemical tests, allows the pattern of carbon sources utilized by bacteria to be determined. The test was prepared and performed in accordance with the manufacturer’s instructions. Incubation was conducted at 30 °C, and the color change of the indicator was observed after 24 and 48 h.

The identification of yeast isolates, which were grown on YGC agar at 25 °C for 5 days (Merck), was carried out microscopically using a wet mount. Unfortunately, we were unable to identify the yeast isolated from the kefir starter cultures. For comparison purposes, a yeast isolate from the pharmaceutical preparation Enterol (Laboratoires Biocodex, Paris, France) was also examined.

Preparation of media: M17 agar and M17 broth, used for the cultivation and multiplication of lactic acid bacteria, were prepared by dissolving powdered M17 medium (Merck) in distilled water in accordance with the manufacturer’s instructions. The prepared media were then autoclaved at 121 ± 1 °C for 15 min. Yeast extract broth (containing 5 g of yeast extract and 10 g of glucose in 1 L of medium, with a pH of 6.5 ± 0.2) was used for the revival and multiplication of yeast isolates. Before their use in the experiments, all bacterial and yeast isolates were revived and multiplied in broth at 30 °C for 24 h.

Cholesterol solution preparation: A cholesterol solution was prepared by dissolving powdered cholesterol with a purity of >99% (Sigma-Aldrich Poland, Poznań, Poland) in a 3:1 mixture of 99% ethanol and Tween 80 (Merck). The dissolution process involved heating the bottle containing the ethanol, Tween 80, and cholesterol mixture in a hot water bath. The final cholesterol concentration of the prepared solution was 40 g/L. Before each cholesterol sample was taken, the solution was heated in a boiling water bath to ensure complete dissolution of the cholesterol crystals.

M17 broth with cholesterol: M17 broth with cholesterol was prepared by adding the sterile cholesterol solution to a sterile basic M17 broth to achieve the desired final cholesterol concentration. All operations were performed under sterile conditions. After thoroughly mixing the contents of the Schott bottles, the broth was used directly in the experiments. Additionally, a control broth sample was prepared for each sample to determine the initial cholesterol concentration in the broth.

Gastric juice model: The gastric juice model was adapted from Clavel et al. [30]. The juice was prepared by dissolving the following reagents in 1000 mL of distilled water: 4.8 g of NaCl (analytical grade, POCH, Gliwice, Poland), 1.56 g of NaHCO_3_ (analytical grade, POCH), 2.2 g of KCl (analytical grade, POCH), and 0.22 g of CaCl_2_ (analytical grade, POCH). The pH of the prepared solution was adjusted to 2.4 ± 0.2 using a pH meter (model LPH330T, Grosseron, SAS, Couëron, France) and a 1 M HCl solution (analytical grade, POCH). The gastric juice was then dispensed into 50 mL Schott bottles and 10 mL test tubes and sterilized in an autoclave at 121 ± 1 °C for 15 min. Immediately before use, a sterile pepsin solution was added to the gastric juice model, as described below.

Pepsin: A lyophilized pepsin preparation (Sigma-Aldrich) with an activity rate of 3200–4500 units per 1 mg of protein was used in the experiments. Pepsin was added directly before the experiments. For this purpose, 2 mg of crystalline pepsin was weighed on a torsion balance (type PRLT T2, Techniprot, Pruszków, Poland) and dissolved in 10 mL of model gastric juice. This solution was then transferred to 50 mL of the gastric juice model and used immediately in the experiments.

The intestinal juice model was prepared based on the method described by Marteau et al. [31], with some modifications. First, a 1 M NaHCO_3_ solution was prepared by dissolving 84.01 g of NaHCO₃ (analytical grade, POCH) in 1000 mL of distilled water. Then, 2.5 g of NaCl (analytical grade, POCH), 0.3 g of KCl (analytical grade, POCH), 0.015 g of CaCl_2_ (analytical grade, POCH), and 8.5 g of ox bile (Merck) were weighed and dissolved in 500 mL of the previously prepared 1 M NaHCO_3_ solution. The pH was adjusted to 7.0 ± 0.2 after sterilization using a pH meter (model pHT 003, Eon Trading LLC, Wilmington, DE, USA) and 1 M NaOH (analytical grade, POCH). The prepared solution was distributed into Schott bottles in 160 mL portions and autoclaved at 121 ± 1 °C for 15 min. A sterile pancreatic enzyme solution was added to the intestinal juice model immediately before use.

Pancreatic enzymes, consisting of a mixture of lipase, amylase, and protease, were used in the pharmaceutical preparation form Kreon^®^ 10,000 (Solvay Pharmaceuticals GmbH, Hanover, Germany). One capsule of this preparation contains the active substance of 150 mg of pancreatin, derived from the bovine pancreas. Its activity rate is 10,000 FIP units of lipase, 8000 FIP units of amylase, and 600 FIP units of protease. The enzymes were added immediately before use by aseptically pouring the contents of 5 capsules into 160 mL of the previously prepared model intestinal juice. This mixture was then placed in a water bath at a temperature not exceeding 35 ± 1 °C for about 10 min to ensure proper dissolution of the Kreon^®^ 10,000 granules, after which it was used directly in the experiments.

For each experiment, a fresh, uniform batch of the gastric or intestinal juice model containing the appropriate enzymes and cholesterol supplement was prepared.

### 2.2. Cholesterol Binding in Culture Medium and During Passage Through Digestive Juice Models

The ability of lactic acid bacteria and yeasts to remove cholesterol from the culture medium, yeast broth, simulated gastric juice, or simulated intestinal juice was assessed by measuring the cholesterol concentration using the method described in Section 2.4 before and after 24 h of incubation at 36.6 °C. Before the actual cholesterol-binding study, the lactic acid bacteria and yeast biomass were precultured at 30 °C for 24 h in M17 broth, yeast extract, or simulated digestive juice models supplemented with a cholesterol solution. The cholesterol concentration in the culture supernatant was determined at the beginning and end of the incubation period using the method described below. The cholesterol concentration was measured using the Cholésterol RTU^®^ enzymatic diagnostic test (bioMérieux, Craponne, France), which provides linear readings over a cholesterol concentration range of 0.081 g/L to 6.97 g/L. Absorbance measurements were performed using a SmartSpecTM 3000 spectrophotometer (Bio-Rad Polska Sp. z.o.o., Warsaw, Poland) at a wavelength of 500 nm. Before determining the degree of cholesterol binding, the contents of the tubes were centrifuged in an ultracentrifuge (12,000× *g*, 10 min, 4 °C) to separate the microbial biomass and obtain a clear culture supernatant. The cholesterol concentration measurements obtained from the media after incubation at 36.6 °C were used to calculate the percentage of cholesterol removed by each of the isolates tested. For each isolate, five experiments were performed. The percentage of bound cholesterol was calculated using the following Formula (1):

(1) $$\mathrm{Percentage}\;\mathrm{of}\;\mathrm{bound}\;\mathrm{cholesterol}\;(\%)=\frac{\left(\mathrm{initial}\;\mathrm{cholesterol}\;\mathrm{concentration}\;-\;\mathrm{final}\;\mathrm{cholesterol}\;\mathrm{concentration}\right)\;}{\mathrm{initial}\;\mathrm{cholesterol}\;\mathrm{concentration}}\times 100\;[g/L]$$

where the initial cholesterol concentration refers to the cholesterol concentration in the medium before the addition of bacterial or yeast cultures, and the final cholesterol concentration refers to the concentration after incubation with the cultures.

### 2.3. Release of Bound Cholesterol During Passage Through Digestive Juice Models

The cellular biomass with bound cholesterol remaining after centrifugation of the culture medium was transferred to an equivalent volume of the gastric juice model, followed by transfer to the intestinal juice model. In the gastric juice model, the biomass was incubated for 2 h at 36.6 °C, and in the intestinal juice model, it was incubated for 5 h at 36.6 °C. After each incubation, the cholesterol concentration in the medium was measured again using the method described in Section 2.4. This experimental setup was designed to investigate the release of cholesterol bound to the lactic acid bacteria and yeast cells. For each isolate, five experiments were performed.

### 2.4. Determination of the Cholesterol Concentration in the Culture Medium

The described enzymatic method was employed to quantify the cholesterol concentration in the culture medium. For this purpose, the Cholésterol RTU^®^ reagent (bioMérieux), commonly used in clinical diagnostics for measuring the total cholesterol in human serum and plasma, was utilized. According to the manufacturer’s specifications, the measurement results exhibit a linear relationship within the cholesterol concentration range of 0 to 6.97 g/L. To determine the cholesterol concentration, the culture media were first transferred to centrifuge tubes and then placed in an ultracentrifuge (Type 317a, Mechanika Precyzyjna, Warsaw, Poland) to separate the cellular biomass and obtain a clear supernatant. Centrifugation was performed for 7 min at 12,000× *g* at 4 ± 1 °C. Measurements were conducted by pipetting 20 μL of the supernatant and 2 mL of the Cholésterol RTU^®^ reagent into test tubes using an automated pipette. Additionally, two control samples were prepared: a cholesterol standard and a reagent blank. The cholesterol standard sample required 20 μL of Cholésterol calibrateur cholesterol standard to be mixed with 2 mL of the Cholésterol RTU^®^ reagent, while the reagent blank consisted of 2 mL of the Cholésterol RTU^®^ reagent alone. After thorough mixing and incubation for 10 min at 20 to 25 °C, the absorbance was measured at a wavelength of 500 nm using a spectrophotometer (Helios Gamma, Thermo Scientific™, Warsaw, Poland) in duplicate for each sample. The results were zeroed against the reagent blank. The cholesterol concentration (in g/L) was calculated using the following Formula (2).

(2) $$\mathrm{Cholesterol}\;\mathrm{concentration}\;\mathrm{in}\;\mathrm{the}\;\mathrm{sample}=\frac{\mathrm{Absorbance}\;\mathrm{measured}\;\mathrm{for}\;\mathrm{the}\;\mathrm{sample}}{\mathrm{Absorbance}\;\mathrm{measured}\;\mathrm{for}\;\mathrm{the}\;\mathrm{cholesterol}\;\mathrm{standard}}\;[g/L]\;$$

An enzymatic method using the Cholésterol RTU^®^ diagnostic reagent (bioMérieux), which includes a set of enzymes, was employed. Cholesterol was determined through the following sequence of enzymatic reactions: (1) cholesterol esterase hydrolyzes cholesterol esters into free cholesterol and fatty acids; (2) cholesterol oxidase oxidizes free cholesterol to cholest-4-ene-3-one and hydrogen peroxide (H_2_O_2_); and (3) peroxidase catalyzes the coupling of H_2_O_2_ with 4-aminoantipyrine (4-AAP) and phenol to produce a quinoneimine chromogen. The intensity of the resulting pink color, which is proportional to the quinoneimine concentration, was measured spectrophotometrically at a wavelength of 500 nm. This color intensity is directly proportional to the cholesterol content in the sample. The enzymatic reaction yields a stable pink color that remains stable for 1 h at 20–25 °C after reagent mixing. A cholesterol concentration standard was used for the determination.

The cholesterol standard (Cholesterol Calibrator, bioMérieux) was produced using stabilized bovine lipoproteins. The ratio of free cholesterol to esterified cholesterol was approximately equivalent to that found in human serum, and the total cholesterol concentration was 5.17 mmol/L (or 2 g/L). In accordance with the manufacturer, the concentration was determined based on SRM 909b (two levels)—the Standard Reference Materials from the National Institute of Standards and Technology (NIST).

### 2.5. Determination of the Numbers of Bacterial and Yeast Cells Using the Plate Method

The number of bacterial cells in the cultures was determined using the pour plate method, using two parallel replicates. The numbers of lactic acid bacteria and yeasts were determined separately using M17 and YGC agar, respectively. Sterile peptone water was used to prepare decimal dilutions. The inoculated plates were incubated at 30 ± 1 °C for 72 h. The results are expressed in colony-forming units per milliliter (CFU/mL) of culture.

### 2.6. Determination of the Biomass Yields of Bacterial and Yeast Cell Cultures

The biomass yields of the bacterial and yeast cell cultures were first determined by the drying method following centrifugation of a defined volume of culture medium in an ultracentrifuge (Mechanika Precyzyjna Warszawa, Type 317a, Warsaw, Poland). After 20 h of incubation in M17 or yeast broth, the cholesterol depletion was measured, and the cell biomass yield was determined gravimetrically (in grams of dry matter per liter, g DM/L). Centrifugation was carried out for 7 min at 12,000× *g* at room temperature. The obtained biomass was then washed twice with sterile, distilled water to remove noncovalently bound cholesterol. Subsequently, the recentrifuged biomass was dried at 80 ± 1 °C until a constant weight was achieved. Biomass drying was carried out at atmospheric pressure. The results are expressed in grams of dry matter per milliliter of liquid culture (g DM/mL). This determined the amount of cholesterol covalently bound by the cells per unit of biomass dry matter content.

### 2.7. Statistical Analysis

The cholesterol depletion measurements and the number of lactic acid bacteria and yeast cells in M17 broth, yeast broth, and under simulated digestive juice conditions with the addition of cholesterol solution were analyzed with the Statgraphics XVII program (Statgraphics Technologies, Inc., The Plains, VA, USA). Significance was defined as α = 0.05.

## 3. Results

### 3.1. Binding of Cholesterol by the Lactococcus Cultures in M17 Broth and During Passage Through Simulated Digestive Juice Models

**Figure 1:**
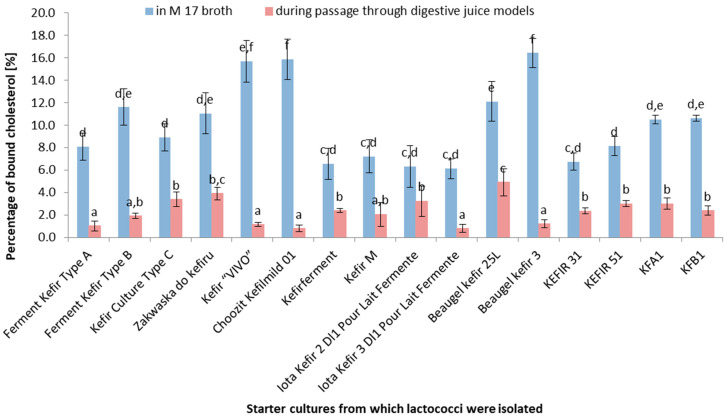
Percentage of bound cholesterol in M17 broth and during simulated digestive juice passage [%] (*n* = 5). ^a–f^—Means with different uppercase letters in the same column are significantly different (*p* < 0.05).

The *Lactococcus* strains showed significantly higher cholesterol-binding percentages in the M17 broth compared to under simulated digestive conditions (Figure 1, *p* < 0.05). The highest cholesterol-binding percentage in the M17 broth was achieved by the *Lactococcus* strains isolated from “Choosit Kefimild D1” and “Beaugel Kefir 3” cultures, while the lowest binding percentage was observed for the “KFA1” culture. Notably, some starter cultures exhibited a substantial difference in their cholesterol-binding percentages between the two conditions (e.g., *Lactococcus* strains isolated from “Ferment Kefir Type A”, “Choosit Kefimild D1”, and “Beaugel Kefir 3”). Other cultures displayed smaller differences between these conditions, suggesting that they are more stable in the presence of digestive juices (e.g., “KEFIR 31”, “KFA1”). These findings indicate that different kefir cultures have varied cholesterol-binding capacities under both laboratory conditions (M17 broth) and simulated digestive conditions. It is notable that under actual digestive conditions (simulated by digestive juice models), the cholesterol-binding abilities of these cultures may be lower. These results may also warrant further research on the stability of kefir cultures in the gastrointestinal tract and their impact on human health.

Table 2 presents the populations of *Lactococcus* cells in different kefir starter cultures at three stages: the initial population of live cells, the final population of live cells in M17 broth after 24 h of incubation at 36.6 °C, and the final population of live cells during simulated digestive juice passage after 7 h at 36.6 °C. It is notable that the final pH of the postculture fluids after M17 incubation was 3.59–4.01, while the final pH in the digestive juice models was slightly higher, 4.15–4.60. The initial population of live cells for all starter cultures was approximately 7.8–7.9 log(CFU/mL). The final population of live cells in M17 broth after 24 h of incubation increased to approximately 8.6–8.7 log(CFU/mL) for all cultures. The increased cell population in the M17 broth indicates the ability of these cultures to multiply under favorable growth conditions. All cultures showed a significant (*p* < 0.05) decrease in the number of live cells during their passage through the digestive juice models compared to their growth in the M17 broth. Under conditions simulating digestive juice passage, the final population of live cells decreased significantly for all starter cultures, reaching 0.1–0.5 log(CFU/mL). The highest survival rate in the digestive juice models was shown by the *Lactococcus* strains isolated from the “Ferment Kefir Type A” and “Ferment Kefir Type B” cultures (0.4–0.5 log(CFU/mL)), while the lowest was obtained for the *Lactococcus* strain isolated from “Iota Kefir 2 Dl1 Pour Lait Fermente” (0.1 log(CFU/mL)). The differences in survival between the cultures may suggest differences in their resistance to digestive conditions, which is important for their potential application in probiotic products.

**Figure 2:**
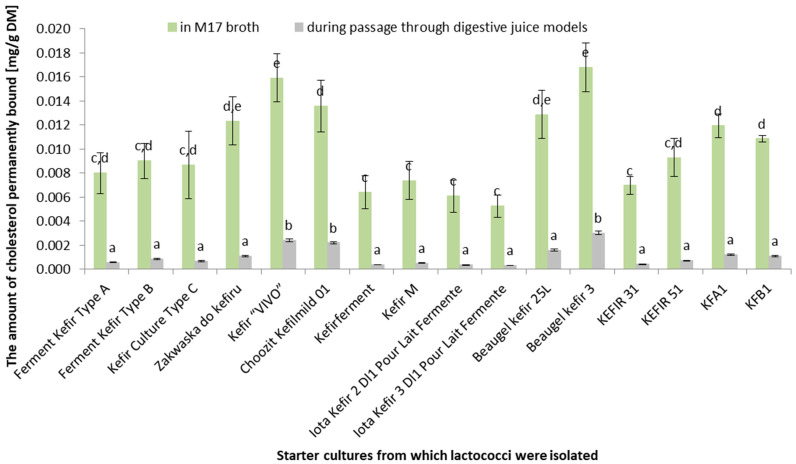
Amount of cholesterol permanently bound in M17 broth and during simulated digestive juice passage [%] (*n* = 5). ^a–e^ Means with different uppercase letters in the same column are significantly different (*p* < 0.05).

Figure 2 illustrates the amount of cholesterol permanently bound by the *Lactococcus* strains isolated from different kefir starter cultures under two conditions: in M17 broth (green) and during simulated digestive juice passage (gray). The *y*-axis shows the amount of permanently bound cholesterol in mg/g DM. The amount of permanently bound cholesterol is significantly (*p* < 0.05) higher in the M17 broth than during simulated digestive juice passage for all investigated starter cultures. The highest amount of permanently bound cholesterol in M17 broth was achieved by the *Lactococcus* strains isolated from the “Choosit Kefimild D1” and “Beaugel Kefir 3” cultures, while the lowest amount was noted for the *Lactococcus* strains isolated from the “KFA1” culture. Some starter cultures showed a significant difference in the amounts of permanently bound cholesterol obtained under the two conditions (e.g., *Lactococcus* strains isolated from “Choosit Kefimild D1” and “Beaugel Kefir 3”). Other cultures showed smaller differences between these conditions, which may suggest that they are more stable in digestive juices (e.g., *Lactococcus* strains isolated from “KEFIR 31” and “KFA1”). The data suggest that different kefir cultures have varied abilities to permanently bind to cholesterol under both laboratory conditions (M17 broth) and simulated digestive conditions. It is notable that under actual digestive conditions (simulated by digestive juice models), the ability to permanently bind to cholesterol may be lower. These results indicate the need for further research on the stability of kefir cultures in the gastrointestinal tract and their impact on human health.

Cultures exhibiting a high cholesterol-binding capacity in M17 broth often display reduced binding abilities under simulated digestive juice passage. This could be due to several factors, such as the acidic pH of gastric juice, the presence of digestive enzymes that degrade the bacterial cell components responsible for cholesterol binding, or competition for cholesterol binding from other food components present in the intestine. For instance, the *Lactococcus* strain isolated from “Choosit Kefimild D1” exhibits one of the highest cholesterol-binding capacities in M17 broth, but this ability significantly declines in digestive models. There is no direct correlation between the cholesterol-binding capacity and survival in digestive models. Cultures with high survival rates in digestive models (e.g., *Lactococcus* strains isolated from “Ferment Kefir Type A” and “Ferment Kefir Type B”) do not necessarily possess the highest cholesterol-binding capacities. The selection of appropriate starter cultures for health-promoting kefir products should consider both the cholesterol-binding ability and survival in the gastrointestinal tract. Cultures exhibiting a moderate cholesterol-binding capacity and a high survival rate may be more effective for practical applications. Kefir cultures with both a cholesterol-binding capacity and a high survival rate may confer greater health benefits, as they have a higher chance of surviving and exerting a beneficial effect in the gastrointestinal tract. Further research could elucidate the mechanisms influencing the cholesterol-binding capacity and survival rate under digestive conditions, as well as aiding in the identification of starter cultures with optimal health-promoting properties.

### 3.2. The Binding of Cholesterol by Yeast Cultures in Yeast Broth and Simulated Digestive Juice Passage

**Figure 3:**
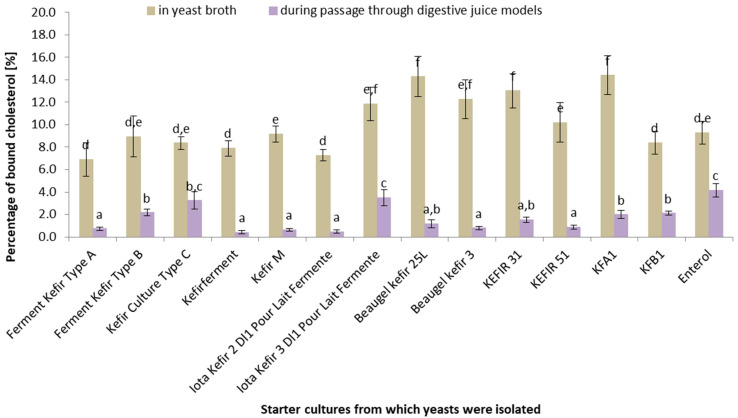
Percentage of bound cholesterol in yeast broth and simulated digestive juice passage [%] (*n* = 5). ^a–f^ Means with different uppercase letters in the same column are significantly different (*p* < 0.05).

Figure 3 illustrates the percentage of cholesterol bound by yeasts isolated from different kefir starter cultures in yeast broth (beige) and during digestive juice passage (purple). Data for different kefir starter cultures are presented on the *x*-axis, and the bound cholesterol percentages are shown on the *y*-axis. Notably, more cholesterol was bound in yeast broth than during digestive juice passage for all investigated starter cultures. The highest cholesterol-binding rate in yeast broth was achieved by the yeasts isolated from Beauge/kefir 3 and KEFIR 31 cultures (around 18–19%), while the lowest rate was exhibited by the yeasts isolated from “Iota Kefir 2 D1” and “Iota Kefir 3 D1 Pour Lait Fermente” cultures (around 8%). Selected yeasts isolated from kefir cultures showed statistically significant differences (*p* < 0.05) in their ability to bind to cholesterol under both conditions. For the digestive juice models, cholesterol binding was most effective for the “Enterol” culture (around 6%), while the yeasts isolated from the “Iota Kefir 2 D1” and “Iota Kefir 3 D1 Pour Lait Fermente” cultures showed the lowest percentage (around 2%). The simulated digestive juice passage significantly reduced the percentage of bound cholesterol, suggesting that digestive conditions significantly impact the ability of cultures to bind to cholesterol. This may indicate that kefir cultures with higher cholesterol-binding rates may be valuable in the context of a cholesterol-lowering diet.

Table 3 presents the yeast cell population during cultivation in yeast broth and simulated digestive juice passage. The highest initial live cell population was observed in the “Enterol” culture (6.7 log(CFU/mL)), while the lowest was observed in the “Kefir Culture Type C” culture (4.1 log(CFU/mL)). Most yeast cultures exhibited significant population growth in yeast broth, suggesting that effective multiplication had occurred under favorable laboratory conditions. The largest increase was observed for yeasts isolated from the “Kefir Culture Type C” culture (8.7 log(CFU/mL)). Yeasts isolated from “Ferment Kefir Type A”, “Ferment Kefir Type B”, “Kefir M”, “Beaugel Kefir 3”, and “Enterol” reached similar final values (*p* < 0.05). A significant decrease in the population of live cells was noted after 7 h in the digestive juice models. Notably, the final pH of the postculture fluids after yeast broth incubation was 4.14–4.21, while that of the digestive juice models was slightly higher, 4.25–4.74. Simulated digestive juice passage significantly reduces the live cell population, indicating the low survival rates of these yeasts. This is important for evaluating the potential efficacy of probiotics, as the low survival rates may limit their health benefits. The highest final live cell population was 0.4 log(CFU/mL) for the yeasts isolated from the “Ferment Kefir Type B” culture. In many cases (e.g., yeasts isolated from “Iota Kefir 2 D1 Pour Lait Fermente” and “Enterol” cultures), almost no live cells were detected (0.0 log(CFU/mL)).

**Figure 4:**
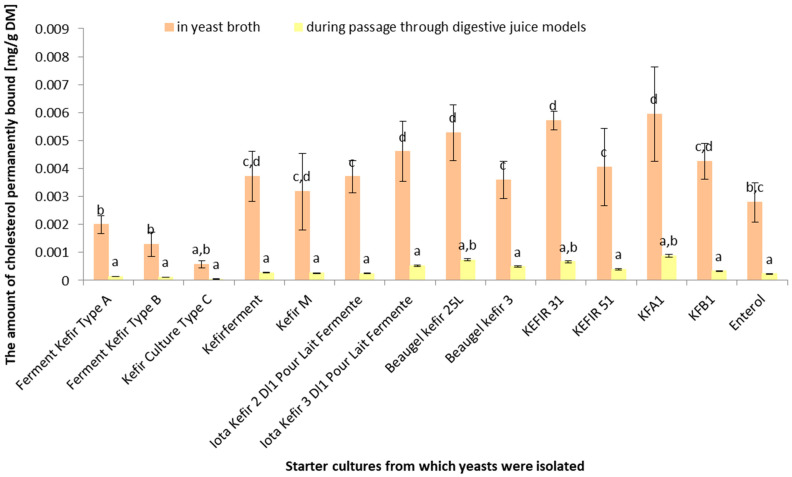
Amount of permanently bound cholesterol in yeast broth and simulated digestive juice passage [%] (*n* = 5). ^a–d^ Means with different uppercase letters in the same column are significantly different (*p* < 0.05).

Figure 4 presents the amount of permanently bound cholesterol (mg/g DM) for different yeast strains isolated from kefir starter cultures. There were statistically significant differences (*p* < 0.05) in the ability of yeast cultures to permanently bind to cholesterol in both yeast broth and during simulated digestive juice passage. The highest amount of bound cholesterol in yeast broth was observed for the yeasts isolated from the “Beaugel Kefir 3” and “KEFIR 51” cultures, about 0.006 mg/g DM. The lowest amount was recorded for the yeasts isolated from the “Kefir Culture Type C” culture, about 0.001 mg/g DM. The values of bound cholesterol during the simulated digestive juice model passage were significantly lower compared to those from yeast broth, around 0.0001 mg/g DM for most cultures. Yeasts isolated from the “KFA1” culture had relatively high, but not significantly different, values compared to other cultures.

A higher initial yeast population may positively impact cholesterol-binding ability, but this suggestion is not conclusive. Other factors may also influence the cholesterol-binding capacity. The highest initial live cell population occurred in the “Enterol” culture (6.7 log(CFU/mL)), which exhibited the highest cholesterol binding during simulated digestive juice passage (approximately 6%). The lowest initial live cell population occurred in the “Kefir Culture Type C” culture (4.1 log(CFU/mL)), which showed the least cholesterol binding in yeast broth (approximately 0.001 mg/g DM).

There is no clear correlation between yeast population growth and cholesterol-binding ability. This ability may depend on the specific properties of each yeast culture. The largest population increase was observed in the “Kefir Culture Type C” culture (from 4.1 to 8.7 log(CFU/mL)); however, this culture had the least permanently bound cholesterol in yeast broth (approximately 0.001 mg/g DM). Cultures that exhibited similar population growth levels (e.g., “Ferment Kefir Type A”, “Ferment Kefir Type B”, “Kefir M”, “Beaugel Kefir 3”, and “Enterol”) had different cholesterol-binding capacities.

There was no direct relationship between yeast survival and the ability to bind to cholesterol under digestive conditions. Other factors may have a greater influence on this ability. Yeasts from the “Enterol” culture underwent the most cholesterol binding during simulated digestive juice passage (approximately 6%) as well as low survival rates (0.0 log(CFU/mL)). Ferment Kefir Type B cultures had the highest final live cell population (0.4 log(CFU/mL)) but did not exhibit the most cholesterol binding.

Differences in the ability to permanently bind cholesterol were more pronounced in yeast broth than in digestive juice models. In yeast broth, the highest amount of permanently bound cholesterol was observed in yeasts from the “Beaugel Kefir 3” and “KEFIR 51” cultures (approximately 0.006 mg/g DM), while the lowest was in the “Kefir Culture Type C” culture (approximately 0.001 mg/g DM). In digestive juice models, the values were significantly lower and closer together (approximately 0.0001 mg/g DM for most cultures).

## 4. Discussion

This study demonstrates a significant difference in the cholesterol-binding levels of *Lactococcus* strains in M17 broth (laboratory conditions) and simulated digestive juice models. Specifically, cholesterol-binding percentages were consistently higher in the M17 broth across all tested strains (*p* < 0.05), indicating that laboratory conditions are more conducive to cholesterol binding than the more challenging digestive environment. While some strains excel in controlled laboratory environments, their efficacy is reduced under digestive conditions due to factors such as the acidic pH and the presence of digestive enzymes in the simulated digestive model [22]. Certain strains, such as those from “Ferment Kefir Type A”, “Choosit Kefimild D1”, and “Beaugel Kefir 3”, displayed substantial differences in cholesterol-binding levels between the two conditions, indicating their reduced stability in digestive environments. Conversely, strains from “KEFIR 31” and “KFA1” showed smaller differences, suggesting their greater resilience to digestive conditions. These findings underscore the importance of considering both laboratory and simulated digestive conditions when evaluating probiotic strains for their cholesterol-binding capabilities. Moreover, our research confirms the observations of other researchers regarding the cholesterol-removing abilities of lactic acid bacteria (including *Lactococcus*) under in vitro conditions [2,3,4]. Albano et al. [32] isolated *Lactococcus lactis* ssp. lactis strains from traditional Italian cheeses that exhibited significantly higher cholesterol-binding capabilities than those observed in our study. The survival rates obtained for the digestive models highlight the need to select strains with both a high cholesterol-binding capacity and resilience to digestive conditions. This dual criterion is critical for developing effective probiotic formulations. The initial populations of *Lactococcus* strains were similar across all cultures (around 7.8–7.9 log(CFU/mL)). After 24 h in M17 broth, populations increased significantly to approximately 8.6–8.7 log(CFU/mL), indicating their robust growth under optimal conditions. There were significantly lower live cell populations during the simulated digestive model passage, with final populations of 0.1 to 0.5 log(CFU/mL). This is consistent with other research, highlighting the challenges of maintaining probiotic viability and functionality during gastrointestinal transit.

*Lactococcus* bacteria, including *Lactococcus lactis*, lower cholesterol levels through various mechanisms [22,23]. Similar to *Lactobacillus*, the *Lactococcus* species are being investigated for their potential as cholesterol-lowering agents [4,5,6,7,8,9]. While the mechanisms involved may vary among different bacterial species and strains, several studies have documented *Lactococcus* interactions with cholesterol. Certain *Lactococcus* strains can bind to cholesterol directly via their cell walls [4]. This mechanism, akin to that observed in some *Lactobacillus* strains, may involve the affinity of cholesterol molecules for specific cell wall components.

Similarities in the binding of aflatoxin B1 (AFB1) by lactic acid bacteria cells [33,34,35] are suggestive of cholesterol binding via the cell wall. Numerous studies have reported AFB1 binding by both live and dead cultures of lactic acid bacteria and bifidobacteria, even though these bacteria cannot metabolize AFB1 [35,36,37,38]. Peltonen et al. [39] examined various lactic acid bacteria cultures and found that all cultures bound to AFB1, albeit at different levels, suggest that these differences stem from variations in the cell wall composition. Lahtinen et al. [35] and Haskard et al. [37] demonstrated that peptidoglycan, a component of bacterial cell walls, is responsible for AFB1 binding and that AFB1 binding is independent of the lactic acid bacteria’s ability to produce exopolysaccharides. Lee et al. [33] showed no correlation between the hydrophobicity of lactic acid bacteria cell surfaces and their AFB1 binding capacity. They further demonstrated that AFB1 adsorption was directly proportional to the number of bacteria and the specific strain used.

Conversely, the desorption (release) of cholesterol is weaker in heat-inactivated bacterial cells compared to live cultures. It was concluded that heat-killing bacteria alter the bacterial cell surface, exposing additional AFB1 binding sites. Oatley et al. [36] reported that bifidobacteria also bind to AFB1, and the heat inactivation of cells enhances this binding. Moreover, the destruction of cell wall components, such as carbohydrates or proteins, drastically reduces the amount of AFB1 bound by bacterial cells. Similar correlations and phenomena are likely to occur in cholesterol binding. It is conceivable that, like *Lactobacillus*, some *Lactococcus* strains can incorporate cholesterol into their cell membranes [2], which could influence the physical and functional properties of the membrane, including its fluidity. *Lactococcus* strains possess BSH activity, which can deconjugate bile salts in the intestine. Deconjugated bile salts are less soluble and more likely to be excreted in feces, potentially leading to reduced cholesterol reabsorption [24,25,26,27,28]. Kimoto et al. [3] demonstrated that *Lactococcus lactis* subsp. *lactis* biovar *diacetylactis* N7 can remove cholesterol from the medium independently of the physiological state of the cells. The reduction in the cholesterol content in the medium occurred due to its binding by live cells during growth and by dead bacterial cells (subjected to heat death). However, the amount of cholesterol removed by live cells was significantly higher than that attached to dead cells, further confirming the hypothesis of simultaneous cholesterol incorporation into cell membranes and cell wall binding. It can be hypothesized that cholesterol removal from the substrates occurs simultaneously through these two processes. Our research corroborates these findings [3,22]. By measuring the optical density of the culture at 620 nm, Kimoto et al. [3] showed that cholesterol present in the growth medium stimulated the growth of the N7 strain. However, it is important to note that these researchers used a liquid GM17-THIO medium containing an additional 0.2% sodium taurocholate, and bile acids are known to precipitate cholesterol, altering the optical density of the culture medium [40].

Similar to *Lactococcus* strains, yeast cultures exhibited greater cholesterol binding in the yeast extract broth than in the digestive models. However, it should be noted that yeast cultures, like *Lactococcus* strains, showed a decreased ability to bind to cholesterol under simulated digestive conditions. The “Enterol” culture performed the best (around 6%), while the least binding was observed for “Iota Kefir 2 D1” and “Iota Kefir 3 D1 Pour Lait Fermente” (around 2%). The yeast cultures displayed substantial growth in yeast broth, but a significant decrease in live cell populations was noted during the simulated digestive passage. These findings suggest that while yeast cultures can grow effectively under laboratory conditions, their survival and cholesterol-binding capacities under digestive conditions are significantly reduced. The lack of a direct correlation between growth and cholesterol binding highlights the need for a targeted selection of yeast strains for specific probiotic applications. A study by Psomas et al. [18] demonstrates the ability of specific yeast strains, such as *S. cerevisiae* 832 from Feta cheese and *S. cerevisiae* KK1 and *I. orientalis* KK5.Y.1 from infant feces, to assimilate cholesterol in vitro, suggesting that these yeasts could be used as probiotics as they show a preference for cholesterol assimilation in their native yeast broth environments. Yoshida et al. [41] highlighted that the hypocholesterolemic activity of mannan, a component of the yeast cell surface polysaccharide, is influenced by its side-chain structure. Mannans with shorter side chains and lower phosphate contents, such as those possibly encountered in yeast broth, are associated with greater hypocholesterolemic activity. This underscores a structural aspect of yeast-based substrate interactions with cholesterol, which vary depending on the culture media. These studies collectively enhance our understanding of yeast–cholesterol interactions, demonstrating a notable capacity for cholesterol assimilation and binding in native yeast broth environments. Yoshida et al. [41,42] reported that *K. marxianus* YIT 8292 exhibited more potent hypocholesterolemic activity than other yeasts, including *Saccharomyces cerevisiae*. The hypocholesterolemic activity of 81 yeast strains was examined in rats that were fed a high-cholesterol diet (HCD). The hypocholesterolemic activity of the yeasts varied significantly between strains [42], consistent with our findings in vitro. However, our yeast strains isolated from kefir starter cultures exhibited limited survival in the digestive juice model, while the literature suggests better survival rates for probiotic yeast strains. Cordonnier et al. [43] studied the survival of the probiotic yeast *S. cerevisiae* strain CNCM I-3856 in dynamic in vitro human digestive tract models. The strain showed high survival rates in the upper gastrointestinal tract, emphasizing the capability of kefir yeast strains to endure human digestion. Similarly, Rahmani et al. [44] isolated yeasts from Iranian traditional milk kefir samples and evaluated their potential probiotic properties. Three strains of *S. cerevisiae* and one strain of *P. fermentas* demonstrated resistance and survival abilities under gastrointestinal physiological conditions, indicating their potential use as probiotic yeast strains suitable for functional foods. Yusuf et al. [45] identified that the cholesterol-lowering effect of lactic acid bacteria from Indonesian kefir grains is more pronounced when the cells are metabolically active. Although this study centers on lactic acid bacteria, the cooperative nature of kefir’s microbial community, including yeast, implies that a synergistic mechanism could be enhancing cholesterol assimilation in the human digestive system. These investigations into kefir yeasts and their interactions with cholesterol within digestive models hint at their potential for contributing to cholesterol management and the prevention of related conditions. Nonetheless, further direct studies examining kefir yeast strains within human or analog digestive models are needed to fully elucidate these effects.

This study reveals critical insights into the cholesterol-binding capacities and survival rates of *Lactococcus* and yeast cultures under laboratory and simulated digestive conditions. Effective probiotic formulations should consider both the cholesterol-binding capacity and survival rates under digestive conditions. Strains showing smaller differences in performance between laboratory and digestive conditions are more desirable for practical applications. Understanding the mechanisms behind cholesterol binding and survival rates under digestive conditions could enhance the development of probiotics with optimal health benefits.

It is important to acknowledge the limitations of this in vitro study. The exact microbial composition of some of the cultures we used (Table 1) is unknown, which limits our ability to attribute specific effects to individual strains. While we have isolated and identified dominant microbial groups (e.g., *Lactococcus* spp. and yeasts), minor, unidentified microorganisms may have influenced our results. Additionally, the manufacturers do not provide the precise ratio of microbes for commercially available cultures with known general composition, further contributing to the complexity of interpreting our findings. Despite these limitations, we believe that our study provides valuable insights into the potential role of these cultures. The simulated digestive environment, while providing valuable insights, might not fully represent the complexity of the human digestive tract. Further research using more advanced in vitro models, such as those incorporating dynamic conditions and interactions with gut microbiota or in vivo studies, is needed to confirm these findings and fully elucidate the impacts of these kefir cultures on human cholesterol management. Future studies could utilize more advanced in vitro models, such as dynamic models that simulate conditions within the gastrointestinal tract, including pH changes, enzymatic activity, and peristalsis, to provide a more accurate assessment of the survival and activity of kefir cultures under conditions closer to those found in vivo. Moreover, models that account for interactions with the gut microbiota, for example, the Simulator of the Human Intestinal Microbial Ecosystem (SHIME) or a model using a continuous-flow colon, would enable the investigation of the impacts of kefir cultures on the composition and function of the gut microbiome. The use of these advanced models would provide a better understanding of kefir’s mechanisms of action in vivo and its potential health benefits.

## 5. Conclusions

This study demonstrates that the cholesterol-binding capacity of selected kefir starter cultures, including *Lactococcus* strains and yeasts, is significantly reduced under simulated gastrointestinal conditions, highlighting the challenges associated with probiotic delivery. Laboratory conditions are more favorable for cholesterol uptake compared to the challenging digestive environment. We observed significant variability in the cholesterol-binding capacity and survival rates between different *Lactococcus* and yeast strains. Certain strains displayed a marked decrease in performance under digestive conditions, while others remained relatively stable. Effective probiotic formulations targeting cholesterol management must prioritize strains with high cholesterol-binding capacities and good survival rates throughout the digestive tract. Strains showing minimal performance differences between laboratory and digestive conditions are particularly promising candidates. Further investigations utilizing in vitro digestion models that simulate human gastrointestinal conditions, as well as in vivo studies, are crucial for confirming these findings and obtaining a complete understanding of the impact of these kefir cultures on cholesterol metabolism.

Investigations into the mechanisms of cholesterol binding and survival in digestive environments are needed. Studies utilizing human or more advanced in vitro digestive models are crucial to fully understanding the effects of kefir cultures on cholesterol management in vivo. The selection of kefir strains with both a high cholesterol-binding capacity and resilience to digestive conditions is critical for developing effective probiotic formulations. Further research utilizing human or more advanced in vitro digestive models, such as dynamic models incorporating gut microbiota interactions, is needed to validate these findings and gain a comprehensive understanding of the in vivo effects of these kefir cultures on cholesterol management.

## Figures and Tables

**Table 1:** The dairy starter cultures used in the experiments and basic information declared by their manufacturers.

Ref.	Producer	Composition
Ferment Kefir Type A	Institut Rosell, Montreal, Canada	*Lactococcus lactis* subsp. *cremoris*, *L. lactis* subsp. *lactis*, *L. lactis* subsp. *diacetylactis*, *Leuconostoc mesenteroides* subsp. *cremoris*, yeasts
Ferment Kefir Type B		
Kefir Culture Type C		
Enterol^®^	BIOCODEX, Gentilly, France	*Saccharomyces boulardii* CNCM I–745
Zakwaska do kefiru	GAMA–TECH Leszek Marek Krześniak, Warsaw, Poland	*Lactococcus lactis*, *Streptococcus thermophilus*, *Leuconostoc mesenteroides*, *Lactobacillus acidophilus*, *Bifidobacterium lactis*, *Lactobacillus delbrueckii* subsp. *bulgaricus*, *L. lactis* subsp. *lactis*
Kefir “VIVO”		
Choozit Kefilmild 01	DuPont Danisco, Copenhagen, Denmark	*L. lactis* subsp. *lactis*, *L. lactis* subsp. *cremoris*, *L. lactis* subsp. *lactis biovar. diacetylactis*, *L. mesenteroides* subsp. *mesenteroides*, *S. thermophilus*, *L. acidophilus*
Kefirferment	LactoFerm, Brouwland, Beverlo, Belgium	Unidentified lactic acid bacteria and yeasts
Kefir M	Ets. A.COQARD, F Pagny–sur–Moselle, France	Unidentified microflora from kefir grains, including lactic acid bacteria and yeasts
Iota Kefir 2 Dl1 Pour Lait Fermente		
Iota Kefir 3 Dl1 Pour Lait Fermente		
Beaugel kefir 25L		
Beaugel kefir 3		
KEFIR 31	Biochem S.r.l., Roma, Italy	*Lactococcus lactis* subsp. *cremoris*, *Lactococcus lactis* subsp. lactis biovar *diacetylactis*, *Leuconostoc mesenteroides* subsp. *cremoris*, *Lactobacillus delbrueckii* subsp. *bulgaricus*, *Saccharomyces cerevisiae*
KEFIR 51		*Lactococcus lactis* subsp. *lactis*, *Lactococcus lactis* subsp. *cremoris*, *Lactococcus lactis* subsp. lactis *biovar diacetylactis*, *Leuconostoc mesenteroides* subsp. *cremoris*, *Saccharomyces unisporus*
KFA1	MicroMilk S.r.l., Cremosano, Italy	*Streptococcus thermophilus*, *Lactococcus lactis* subsp. *lactis*, *Lactococcus lactis* subsp. *cremoris*, *Leuconostoc mesenteroides* subsp. *cremoris*, *Lactococcus lactis* subsp. *lactis* biovar *diacetylactis*, *Debarymyces hansenii*, *Kluyveromyces marxianus* subsp. *marxianus*
KFB1		*Streptococcus thermophilus*, *Lactococcus lactis* subsp. *lactis*, *Lactococcus lactis* subsp. *cremoris*, *Leuconostoc mesenteroides* subsp. *cremoris*, *Lactococcus lactis* subsp. *lactis* biovar *diacetylactis*, *Debaryomyces hansenii*

**Table 2:** Lactococcal cell population during their culture in M17 broth and passage through digestive juice models.

Final Population During Simulated Digestive Juice Passage (36.6 °C/7 h) [log(CFU/mL)]	Final Population in M17 Broth (36.6 °C/24 h) [log(CFU/mL)]	Initial Population [log(CFU/mL)]	Starter Cultures from Which Lactococci Were Isolated
0.4 ^a^ ± 0.2	8.6 ^b^ ± 0.1	7.8 ^b^ ± 0.1	Ferment Kefir Type A
0.5 ^a^ ± 0.1	8.7 ^b^ ± 0.0	7.9 ^b^ ± 0.1	Ferment Kefir Type B
0.2 ^a^ ± 0.2	8.6 ^b^ ± 0.1	7.9 ^b^ ± 0.1	Kefir Culture Type C
0.2 ^a^ ± 0.2	8.6 ^b^ ± 0.2	7.8 ^b^ ± 0.1	Zakwaska do kefiru
0.2 ^a^ ± 0.2	8.6 ^b^ ± 0.1	7.8 ^b^ ± 0.1	Kefir “VIVO”
0.2 ^a^ ± 0.2	8.7 ^b^ ± 0.1	7.9 ^b^ ± 0.1	Choozit Kefilmild 01
0.3 ^a^ ± 0.1	8.6 ^b^ ± 0.1	7.8 ^b^ ± 0.1	Kefirferment
0.3 ^a^ ± 0.2	8.6 ^b^ ± 0.1	7.8 ^b^ ± 0.1	Kefir M
0.1 ^a^ ± 0.2	8.6 ^b^ ± 0.1	7.8 ^b^ ± 0.1	Iota Kefir 2 Dl1 Pour Lait Fermente
0.4 ^a^ ± 0.2	8.7 ^b^ ± 0.1	7.9 ^b^ ± 0.1	Iota Kefir 3 Dl1 Pour Lait Fermente
0.3 ^a^ ± 0.2	8.6 ^b^ ± 0.1	7.8 ^b^ ± 0.1	Beaugel kefir 25L
0.3 ^a^ ± 0.1	8.6 ^b^ ± 0.1	7.8 ^b^ ± 0.1	Beaugel kefir 3
0.2 ^a^ ± 0.1	8.6 ^b^ ± 0.1	7.8 ^b^ ± 0.1	KEFIR 31
0.3 ^a^ ± 0.0	8.6 ^b^ ± 0.1	7.8 ^b^ ± 0.1	KEFIR 51
0.2 ^a^ ± 0.1	8.6 ^b^ ± 0.1	7.8 ^b^ ± 0.1	KFA1
0.2 ^a^ ± 0.2	8.6 ^b^ ± 0.1	7.8 ^b^ ± 0.1	KFB1

^a,b^—Means with different uppercase letters in the same column are significantly different (*p* < 0.05).

**Table 3:** Yeast cell population during culture in yeast broth and simulated digestive juice passage.

Final Population During Simulated Digestive Juice Passage (36.6 °C/7 h) [log(CFU/mL)]	Final Population in Yeast Broth (36.6 °C/24 h) [log(CFU/mL)]	Initial Population [log(CFU/mL)]	Starter Cultures from Which Yeasts Were Isolated
0.3 ^a^ ± 0.2	8.0 ^e,f^ ± 0.1	4.3 ^b^ ± 0.1	Ferment Kefir Type A
0.4 ^a^ ± 0.1	8.3 ^e,f^ ± 0.1	5.6 ^c^ ± 0.1	Ferment Kefir Type B
0.1 ^a^ ± 0.2	8.7 ^f^ ± 0.2	4.1 ^b^ ± 0.1	Kefir Culture Type C
0.2 ^a^ ± 0.2	7.8 ^e^ ± 0.1	5.6 ^c^ ± 0.1	Kefirferment
0.2 ^a^ ± 0.2	8.0 ^e,f^ ± 0.2	4.3 ^b^ ± 0.1	Kefir M
0.0 ^a^ ± 0.1	7.8 ^e^ ± 0.1	6.0 ^c,d^ ± 0.1	Iota Kefir 2 Dl1 Pour Lait Fermente
0.3 ^a^ ± 0.2	7.9 ^e^ ± 0.2	4.5 ^b^ ± 0.1	Iota Kefir 3 Dl1 Pour Lait Fermente
0.3 ^a^ ± 0.2	7.9 ^e^ ± 0.1	4.1 ^b^ ± 0.1	Beaugel kefir 25L
0.3 ^a^ ± 0.2	8.0 ^e,f^ ± 0.1	4.9 ^b,c^ ± 0.1	Beaugel kefir 3
0.1 ^a^ ± 0.1	7.8 ^e^ ± 0.1	5.5 ^c^ ± 0.1	KEFIR 31
0.2 ^a^ ± 0.1	7.9 ^e^ ± 0.1	4.3 ^b^ ± 0.1	KEFIR 51
0.1 ^a^ ± 0.1	7.9 ^e^ ± 0.1	4.1 ^b^ ± 0.1	KFA1
0.1 ^a^ ± 0.1	7.8 ^e^ ± 0.1	5.5 ^c^ ± 0.1	KFB1
0.0 ^a^ ± 0.1	8.0 ^e,f^ ± 0.1	6.7 ^d^ ± 0.2	Enterol

^a–f^ Means with different uppercase letters in the same column are significantly different (*p* < 0.05).

## Data Availability

The data that support the findings of this study are available from the corresponding author (M.Z.) upon reasonable request.
